# Case report: Cutaneous anthrax diagnosed using mNGS of a formalin-fixed paraffin-embedded tissue sample

**DOI:** 10.3389/fcimb.2024.1329235

**Published:** 2024-04-04

**Authors:** Jin Zhang, Xin-Yi Hou, Jing-Yu Wang, Bin Lu

**Affiliations:** ^1^ Department of Clinical Medicine, Jining Medical University, Jining, Shandong, China; ^2^ Department of Dermatology, Affiliated Hospital of Jining Medical University, Jining, Shandong, China

**Keywords:** metagenomic next-generation sequencing (mNGS), cutaneous anthrax, *Bacillus anthracis*, case report, formalin-fixed paraffin-embedded, FFPE

## Abstract

The metagenomic next-generation sequencing (mNGS) method is preferred for genotyping useful for the identification of organisms, illumination of metabolic pathways, and determination of microbiota. It can accurately obtain all the nucleic acid information in the test sample. Anthrax is one of the most important zoonotic diseases, infecting mainly herbivores and occasionally humans. The disease has four typical clinical forms, cutaneous, gastrointestinal, inhalation, and injection, all of which may result in sepsis or meningitis, with cutaneous being the most common form. Here, we report a case of cutaneous anthrax diagnosed by mNGS in a butcher. Histopathology of a skin biopsy revealed PAS-positive bacilli. Formalin-fixed paraffin-embedded (FFPE) tissue sample was confirmed the diagnosis of anthrax by mNGS. He was cured with intravenous penicillin. To our knowledge, this is the first case of cutaneous anthrax diagnosed by mNGS using FFPE tissue. mNGS is useful for identifying pathogens that are difficult to diagnose with conventional methods, and FFPE samples are simple to manage. Compared with traditional bacterial culture, which is difficult to cultivate and takes a long time, mNGS can quickly and accurately help us diagnose anthrax, so that anthrax can be controlled in a timely manner and prevent the outbreak of epidemic events.

## Introduction

Anthrax is a serious infectious disease caused by gram-positive, rod-shaped bacteria known as *Bacillus anthracis* (Pal et al., 2023). Humans are infected through contacting with diseased or dead animals and their contaminated products. The organism exists in two physical forms, the biologically active vegetative form and the biologically inert spore form. The majority of anthrax (>95%) are cutaneous anthrax, with most lesions occurring on exposed areas of the body such as the hands, arms, face, and neck (Bower et al., 2022). It occurs in people who have frequent contact with livestock, such as herdsmen, veterinarians, and slaughter workers. The lesions are characterized by a group of small blisters or bumps, which may itch (Suggu and Konakanchi, 2021). The lesions tend to ulcerate and swelling. The lesion subsequently becomes a painless skin sore with an eschar (Celik and Gonen, 2021). Skin lesions are often the first symptoms to be noticed. However, the incidence of cutaneous anthrax cases has declined worldwide. In the past 10 years, less than 400 cases of anthrax have been reported in China each year (Liu et al., 2023). Owing to its rarity, many clinicians are unable to make timely diagnosis of cutaneous anthrax based on the characteristics of the skin lesions alone. Routine culture is often negative, owing to the early administration of broad spectrum or prophylactic antimicrobial drugs. Therefore, making a diagnosis is difficult using conventional methods. Metagenomic next-generation sequencing (mNGS) provides a comprehensive analysis of microbial and host genetic material in clinical samples and can detect all culturable and non-culturable pathogens in the host. mNGS is a useful tool for early and accurate identification of pathogens (Yang et al., 2022). We report a case of cutaneous anthrax diagnosed using mNGS.

## Case report

A 67-year-old man presented with a 10-day history of a purplish-red swelling on the skin of the right wrist and a 9-day history of fever. A rice grain-sized purplish-red papule had appeared on the skin of his right wrist with little itchy and had subsequently gradually increased in size. He had fever 39°C and accompanied by chills. He was treated with intramuscular antipyretic medication and his temperature returned to normal. However, the skin lesion continued increased in size, so he came to our department for treatment.

**Figure 1:**
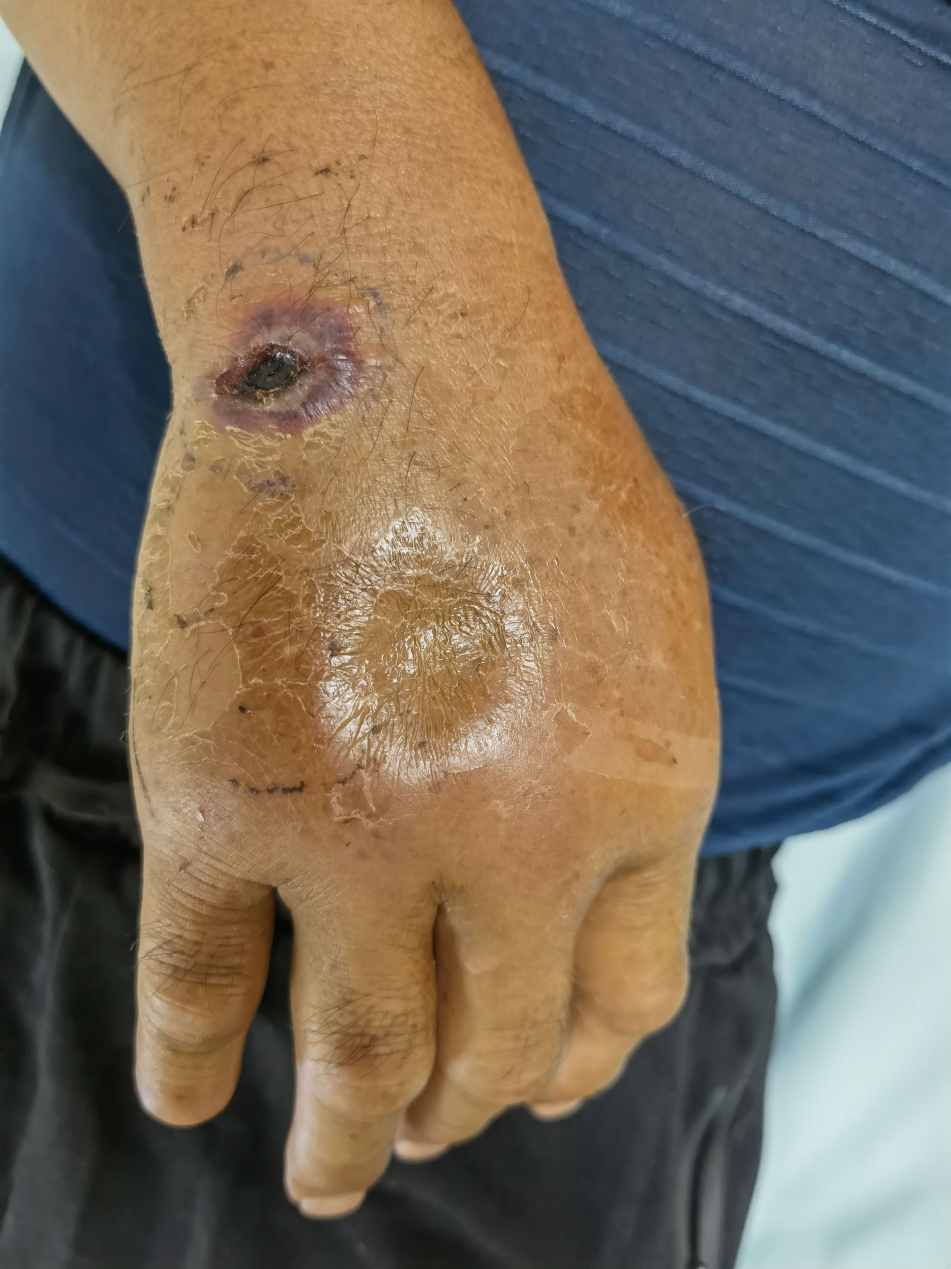
Purple swelling and pitting edema on the back of the right hand.

Physical examination revealed a purple swelling and pitting edema on the right back of the hand, wrist, and forearm; there was ulcer with black eschar on his wrist (**Figure 1**).

The white blood cell count was 9.6 × 109/L, neutrophils was 6.89 × 109/L, and C-reactive protein level was 12.80 mg/L. Liver function and kidney function tests were normal. A *Treponema pallidum* particle agglutination, toluidine red unheated serum test was negative. The syphilis and HIV antibody tests were negative.

**Figure 2:**
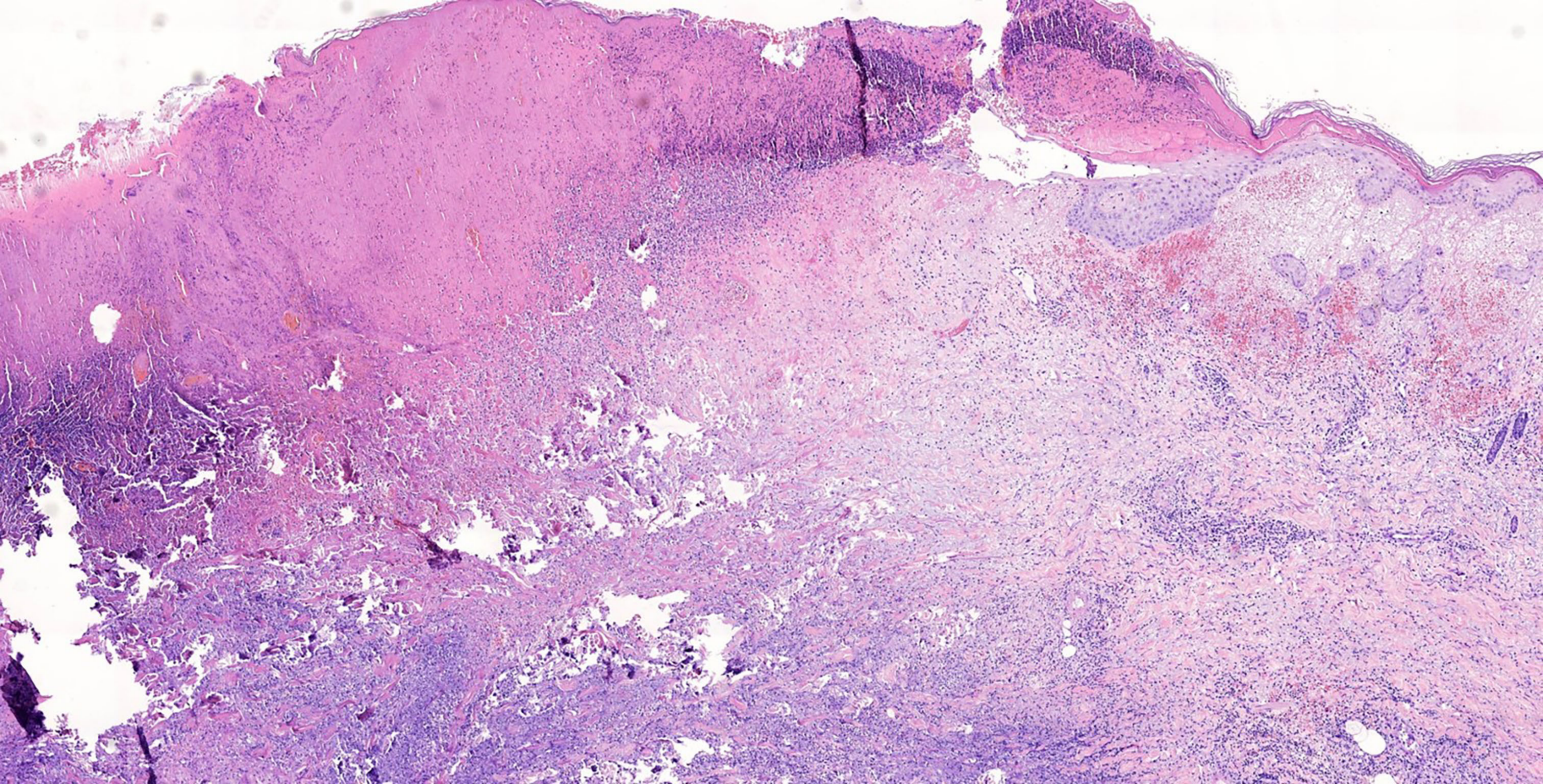
Acute and chronic inflammation with necrosis in the lesions.

Histopathology of skin biopsy showed acute and chronic inflammation with necrosis; combined with Periodic Acid-Schiff stain (PAS) positivity, bacilli clusters were found in the submitted tissue, and acid-fast staining was negative (**Figure 2**).

**Figure 3:**
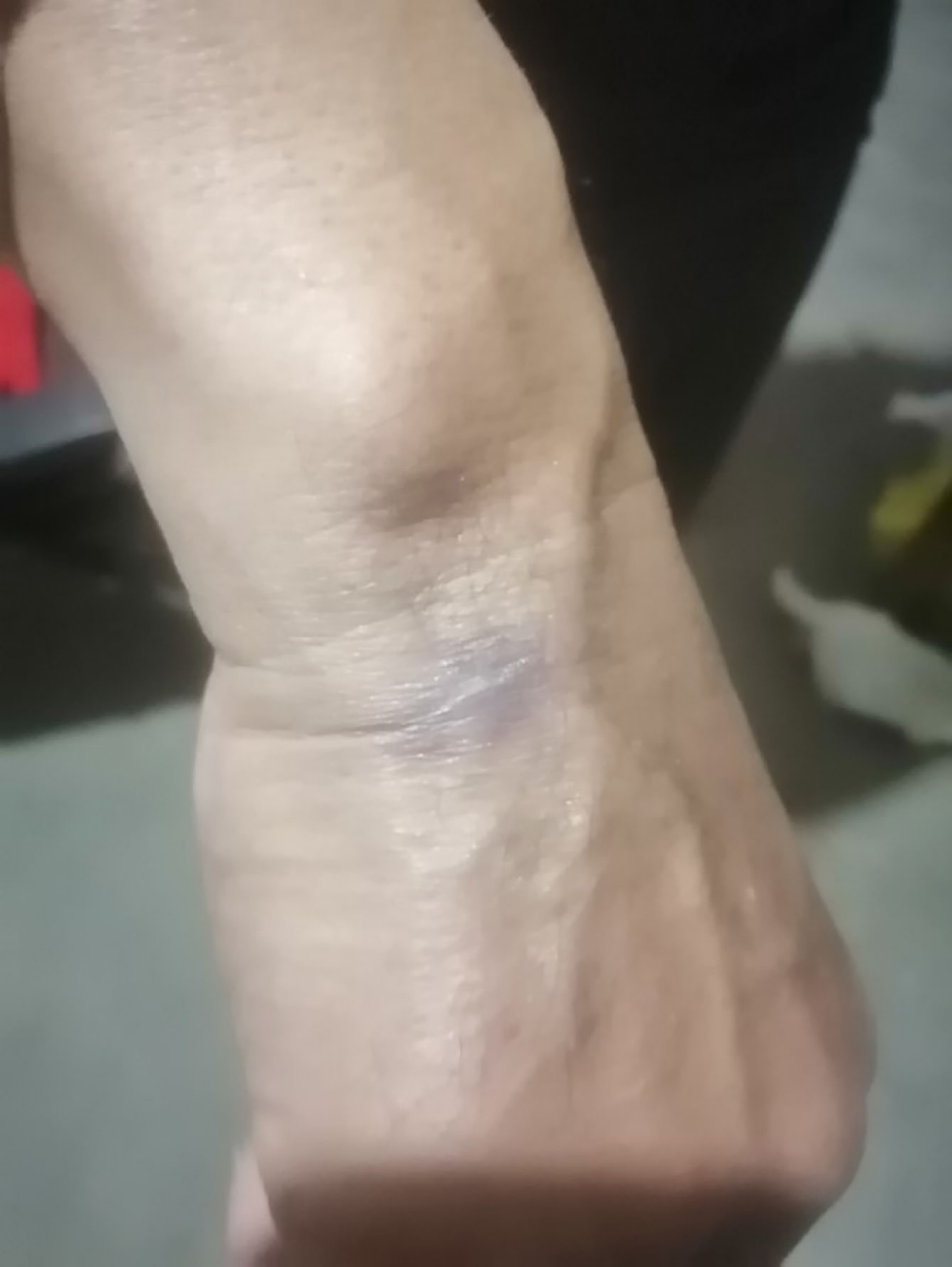
Patient’s swelling subsided, wound healing, scabs off.

The patient was a butcher. He had slaughtered a goat that died of an unknown cause more than 10 days before the disease onset. The histopathology and the patient’s history of contacting with a dead goat led us to suspect cutaneous anthrax. Formalin-fixed paraffin-embedded (FFPE) skin lesion tissue samples were sent to testing company for mNGS to confirm the diagnosis. The mNGS test took only 10h and showed Bacillus anthracis (13,092 reads), accounting for 68.95% of nucleotide sequence coverage (**Table 1**)confirming the diagnosis of cutaneous. The patient was immediately placed in quarantine and treated with intravenous penicillin sodium 2.4 g bid. After starting the treatment, the swelling around the skin lesion subsided, the wound healed, and the scabs fell off 2 weeks later (**Figure 3**). Patient satisfaction and treatment outcomes were excellent.

**Table 1:** mNGS results of the patient.

Genus			Species		
Type	Pathogen	Reads	Pathogen	Reads	Coverage
G+bacteria	Bacillus	13092	Bacillus cereus group	13091	68.95%

## Discussion

Anthrax is one of the most important zoonotic diseases, primarily infecting herbivores and occasionally humans. Infections still occur in low-income areas and occur in humans through contact with sick or dead animals, contaminated animal products, or direct exposure to spores in the environment (Doganay et al., 2023)In our case, the histopathology revealed PAS-positive bacilli. PAS positivity indicates the presence of that bacterial, fungal, or parasitic infections, resulting in abnormal expression of polysaccharides and proteins in cells or tissues. Acid-fast staining was negative, ruling *Mycobacterium tuberculosis* and *Mycobacterium leprae* as possible causes. However, the species cannot be identified based on the above results.

Conventional or culture-based methods usually take 2–3 days for initial results and up to 1 week for confirmation. However, most current mNGS platforms are able to complete analysis from sampling to final results within 24–72h (Han et al., 2019). mNGS can be useful for the identification of bacteria, fungi, viruses, parasites, and other pathogens. Compared with traditional clinical microbial detection methods, mNGS not only effectively improves the detection rate of pathogenic microorganisms but also makes up for the shortcomings of traditional detection methods, especially playing an important role in the precise diagnosis and treatment of some severe infectious diseases that are difficult to diagnose. mNGS can be used to distinguish infection from non-infection, bacterial or viral infection, severity of infection, and probe capture of infection markers (Jiang et al., 2023) By reviewing the literature, we found a case of using fresh blister fluid to perform mNGS to diagnose cutaneous anthrax (Liu et al., 2023). In contrast, we use mNGS on FFPE tissues, which requires less tissue. FFPE tissue samples can be stored for a longer period of time and does not require fresh blister. Specimens are less demanding. So, we successfully diagnosed cutaneous anthrax through mNGS on FFPE tissues, promptly treated the patient with penicillin and achieved a good prognosis. The application of mNGS technology in medicine also has certain limitations, such as complex operations, high costs, and the detection process has not yet been standardized, and in the absence of a unified standard for the mNGS biochemical analysis process, users choose biochemical analysis software based on personal experience and simplicity, which will affect the repeatability of experiments and the reliability of results (Han et al., 2019; Wu et al., 2022). However, there is potential for cost reductions due to increased demand and technological development. As costs decrease, mNGS technology will be more widely used.

## Patient perspective

The patient expressed that he was very satisfied with the treatment effect.

## Conclusion

To our knowledge, this is the first case of cutaneous anthrax to be diagnosed using mNGS of FFPE tissue. mNGS is useful for detecting bacterial, fungal, and viral infections that are difficult to diagnose using conventional methods.

## Data availability statement

The original contributions presented in the study are included in the article/supplementary material. Further inquiries can be directed to the corresponding author.

## Ethics statement

Written informed consent was obtained from the individual(s) for the publication of any potentially identifiable images or data included in this article.

## Author contributions

JZ: Writing – original draft. X-YH: Writing – review & editing. J-YW: Writing – review & editing. BL: Writing – review & editing.
